# In Vitro Anti-SARS-CoV-2 Activity of Selected Metal Compounds and Potential Molecular Basis for Their Actions Based on Computational Study

**DOI:** 10.3390/biom11121858

**Published:** 2021-12-10

**Authors:** Damiano Cirri, Tiziano Marzo, Iogann Tolbatov, Alessandro Marrone, Francesco Saladini, Ilaria Vicenti, Filippo Dragoni, Adele Boccuto, Luigi Messori

**Affiliations:** 1 Laboratory of Metals in Medicine (MetMed), Department of Chemistry “U. Schiff”, University of Florence, Via della Lastruccia 3, 50019 Sesto Fiorentino, Italy; damiano.cirri@dcci.unipi.it; 2 Department of Chemistry and Industrial Chemistry (DCCI), University of Pisa, Via G. Moruzzi 13, 56124 Pisa, Italy; 3 Department of Pharmacy, University of Pisa, Via Bonanno Pisano 6, 56126 Pisa, Italy; 4 Institut de Chimie Moleculaire de l’Université de Bourgogne (ICMUB), UMR CNRS 6302, Université de Bourgogne Franche-Comté (UBFC), Avenue Alain Savary 9, 21078 Dijon, France; 5 Dipartimento di Farmacia, Università “G. d’Annunzio” Chieti-Pescara, Via dei Vestini, 66100 Chieti, Italy; amarrone@unich.it; 6 Department of Medical Biotechnologies, University of Siena, Viale Bracci 16, 53100 Siena, Italy; vicenti@unisi.it (I.V.); filippo.dragoni@unisi.it (F.D.); adele.boccuto@gmail.com (A.B.)

**Keywords:** SARS-CoV-2, COVID-19, antiviral drugs, metallodrugs, gold, ruthenium, antimony, titanium, auranofin, viral infection

## Abstract

Metal-based drugs represent a rich source of chemical substances of potential interest for the treatment of COVID-19. To this end, we have developed a small but representative panel of nine metal compounds, including both synthesized and commercially available complexes, suitable for medical application and tested them in vitro against the SARS-CoV-2 virus. The screening revealed that three compounds from the panel, i.e., the organogold(III) compound Aubipyc, the ruthenium(III) complex KP1019, and antimony trichloride (SbCl_3_), are endowed with notable antiviral properties and an acceptable cytotoxicity profile. These initial findings prompted us to perform a computational study to unveil the likely molecular basis of their antiviral actions. Calculations evidenced that the metalation of nucleophile sites in SARS-CoV-2 proteins or nucleobase strands, induced by Aubipyc, SbCl_3_, and KP1019, is likely to occur. Remarkably, we found that only the deprotonated forms of Cys and Sec residues can react favorably with these metallodrugs. The mechanistic implications of these findings are discussed.

## 1. Introduction

The outbreak and rapid spread of COVID-19, sometimes associated with severe symptoms requiring hospitalization, and, less frequently, with lethal complications, are posing dramatic problems to health systems worldwide [1], with serious consequences to social relationships and economic growth. Although vaccines are now available and have demonstrated high efficacy in decreasing the severity of SARS-CoV-2, reducing hospitalizations and deaths, vaccination does not prevent SARS-CoV-2 transmission [2]. The discovery and rapid implementation of effective antiviral drugs against SARS-CoV-2 would thus represent an extremely important synergistic approach for fighting this pathogen [3]. Indeed, recent evidence indicates that treatment with the nucleoside analogue Molnupiravir reduced the risk of admission to hospital and death in non-hospitalized adults who had mild to moderate COVID-19 symptoms [4]. To this end, expanding the chemical space of the tested compounds by including a variety of metallic compounds is highly desirable. Several gold, bismuth, antimony, and mercury compounds have been used to treat a variety of diseases, mostly infectious ones, including tuberculosis and syphilis, and many parasitic diseases. Even arsenicals, despite being real poisons, were employed in clinics at low doses for various therapeutic purposes with some positive results [5,6,7].

**Figure 1:**
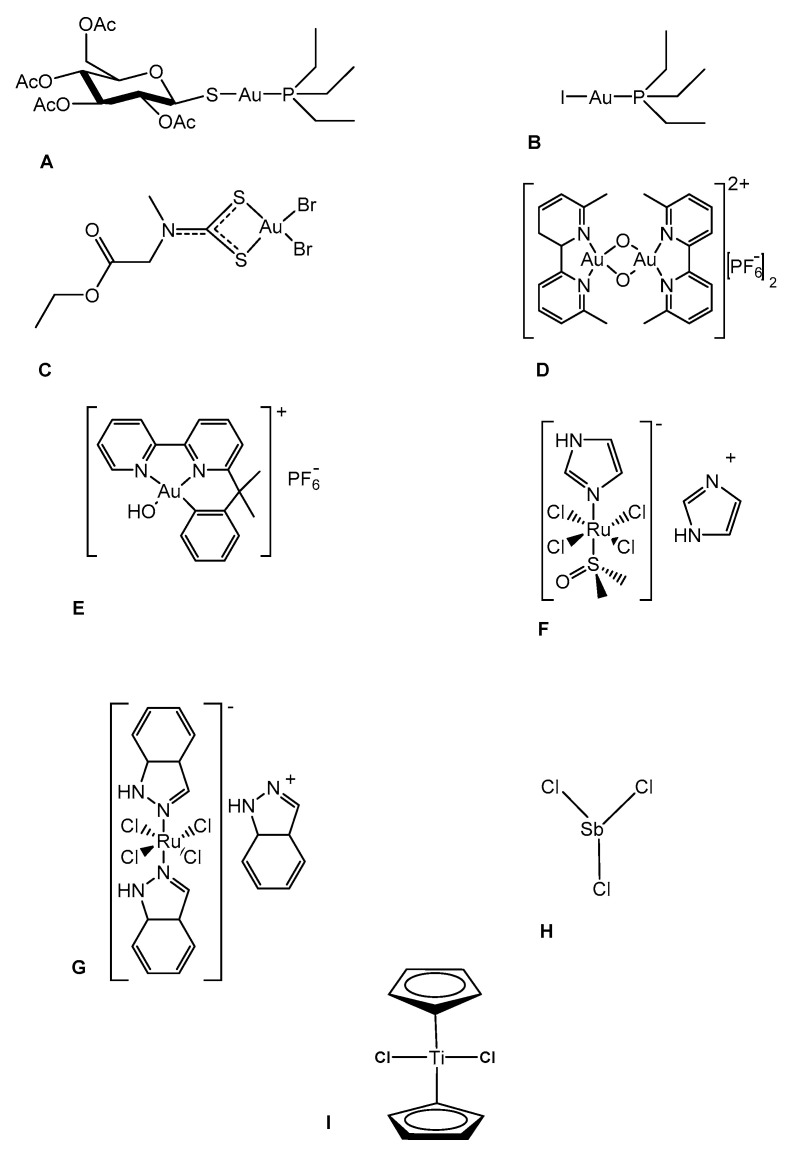
Chemical structures of the investigated complexes: (**A**) Auranofin; (**B**) the iodo-analogue of Auranofin, where the thiosugar is replaced by iodide ligand; (**C**) AuL12; (**D**) AuOXO6; (**E**) Aubipyc; (**F**) NAMI-A; (**G**) KP1019; (**H**) SbCl_3_; (**I**) TiCp_2_Cl_2_ (also known as Titanocene dichloride).

Some inorganic drugs are still in use in current clinical practice for a few specific applications [7,8]. The known inorganic drugs contain a wide array of metals or metalloids imparting specific chemical properties, which arise from the electronic structure of the metal, its coordination sphere, the characteristic of the ligands, the redox properties, etc. It is evident that these chemical features cannot be completely reproduced by simple organic compounds. Accordingly, the unique chemical and biological properties of the various metal (metalloid) centers—in several cases, non-physiological metals—should be considered for medical testing against various disease models [9,10,11,12,13]. This approach might lead to positive pharmacological and therapeutic outcomes, as is the case for several inorganic compounds employed against a variety of diseases (see Figure 1 for examples of inorganic drugs with established or potential medicinal applications).

Generally, metal compounds are believed to exert their cellular and biological effects through the direct inhibition of enzymes; the alteration of transcription factors; their interaction with a variety of biological substrates through coordinative bonding; enhanced lipophilicity; the alteration of cell membrane functions; interference with the cell cycle, and several other key cellular pathways. Medicinally used metal compounds often possess a soft metal center, e.g., gold(I), platinum(II), or silver(I), according to the Pearson HSAB theory, featured by a strong affinity for proteins and enzymes containing accessible and functionally relevant thiol or selenol groups [14,15,16,17]. These arguments support the importance of the systematic exploration of the potential of metal compounds in drug discovery programs for COVID-19 therapeutics. Several representative metal compounds must be considered in the current chemical libraries for screenings. As a matter of fact, over the last year, a few interesting studies concerning inorganic compounds as potential anti-SARS-CoV-2 agents have appeared, with some important results and observations. We refer, for instance, to the studies carried out by various authors on bismuth, gold, and rhenium compounds [18,19,20,21]; moreover, various laboratories—including ours—proposed the clinically established antiarthritic drug Auranofin as an effective antiviral drug candidate through a repurposing strategy [22,23]. More recently, Ingo Ott and coworkers carried out a systematic screening on a large panel, including more than 100 inorganic compounds, for their ability to inhibit the S/ACE2 interaction and the Papain-like Protease PLpro [24]. In any case, no truly effective metal-based drug of straightforward clinical use for COVID-19 treatment has been identified so far. Starting from these considerations, we have prepared a small but representative panel of metal compounds of medicinal interest with the aim of evaluating their efficacy in vitro against SARS-CoV-2. The panel compounds were screened for their anti-SARS-CoV-2 properties according to an experimental protocol established at the University of Siena [25]. As the screening highlighted the favorable anti-SARS-CoV-2 properties of three panel compounds, we decided to perform a computational study to better understand the likely origins of their antiviral properties. The combined experimental and theoretical approach allowed us to unveil some relevant chemical aspects for the action of these metal compounds, which might be advantageously exploited for the design and testing of metallodrugs against SARS-CoV-2.

## 2. Results and Discussion

### 2.1. Construction of the Panel

Owing to our long experience in the field of metal-based drugs, we could quite straightforwardly establish a small panel of representative metal compounds that included many different metal centers, such as ruthenium, gold and titanium [23]. The general criteria that have guided the formation of the panel are the following:

1. A significant chemical diversity, even in the nature of the metal centers.
2. An acceptable stability (i.e., under the applied experimental conditions, panel compounds do not undergo degradation or any other transformation affecting the pharmacological activity, or the interpretation of the results).
3. An acceptable solubility in an aqueous environment.
4. Where possible, an already established role and use in medicinal chemistry.

The chemical structures of the panel compounds are shown in Figure 1.

These compounds are of different origins: some are commercially available, others were previously prepared and characterized. A few, e.g., Auranofin, NAMI-A, KP1019, TiCp_2_Cl_2,_ are clinically established or have entered clinical trials [6,26,27,28,29,30].

### 2.2. Screening of the Panel Compounds for Their Antiviral Properties: The Selection of the Best Drug Candidates

Despite their small size, the compounds on the panel were chosen to ensure a rather large chemical diversity. The panel compounds indeed bear a variety of metal (or metalloid) centers, such as gold, ruthenium, antimony, and titanium. The choice of compounds was driven by chemical and biological considerations (e.g., hard–soft properties of the metal/metalloid center capable of binding specific viral targets; stability; acceptable tolerability/toxicity) [7,23].

First, the investigated compounds were screened to determine their half-maximal cytotoxic concentration (CC_50_) in the Calu-3 cell line model. Once the CC_50_ value had been determined, the highest non-toxic dose was used as the starting drug concentration in the subsequent antiviral assays for each compound. Afterwards, the metal complexes that showed an acceptable cytotoxicity were tested for their antiviral properties against SARS-CoV-2 (Table 1).

The selectivity index (SI) of the three compounds active in the DYRA was 10.6, 6.8 and 6.4 (for Aubipyc, KP1019 and SbCl_3_, respectively). The TiCp_2_Cl_2_ compound, active only in SYRA, had an SI above 4.2. Differently from other active compounds, the observed inhibitory activity of TiCp_2_Cl_2_ in SYRA, but not in DYRA, might indicate a mechanism of action exerted in the late phases of viral replication, e.g., assembly, maturation, and/or the infectivity of viral particles. We did not observe any effect of Auranofin on viral replication, in contrast with previous data, although the cytotoxicity was comparable in both studies [31]. The reasons for this discrepancy might be due to the different cell lines used to evaluate antiviral activity (Calu-3 vs. Huh-7), and the different approaches adopted to measure viral replication (quantification of the expression of viral proteins vs. viral RNA in cell supernatant). In addition, the IC_50_ value measured in Huh-7 cells was close to the CC_50_ value (1.4 µM and 5.7 µM, respectively), indicating that the dose–response curves of antiviral activity and cytotoxicity were almost overlapping.

### 2.3. Mechanistic Studies: The Reactions of the Best Drug Candidates with Selected Biomolecular Targets Analyzed In Silico

The recently characterized RNA genome sequence of SARS-CoV-2 offers the possibility to hypothesize which are the most likely protein targets for effective treatments. The most important are: the spike protein responsible for virus binding to the host cell surface receptor, i.e., angiotensin-converting enzyme 2 (ACE2); coronavirus main proteinase (3CLpro) and papain-like protease (PLpro), which perform the proteolytic cleavage of the polyproteins essential for the production of new mature virions; RNA-dependent RNA polymerase (RdRp), responsible for replicating the RNA genome; and nsp12 polymerase and nsp13 helicase [32,33,34,35]. Currently, there is a lot of interest in uncovering the detailed mechanisms of interaction of antiviral drugs and metallodrugs with the likely biomolecular targets of SARS-CoV-2 [23]. In the present work, based on the above screening procedure, we found that three panel compounds (i.e., Aubipyc, KP1019, and SbCl_3_) possess a quite favorable biological and pharmacological profile. To characterize the possible mechanisms of interaction of these metal compounds with their likely biomolecular targets, we studied the thermodynamics of their interactions with suitable metal-coordinating sites on viral proteins (cysteine Cys, selenocysteine Sec, histidine His) or nucleobase strands (guanine G, adenine A).

In particular, the binding affinities of the selected metal complexes with endogenous nucleophile sites, such as Cys, Cys^−^, Sec, Sec^−^, His, and the nucleobases guanine and adenine, were estimated. Both neutral and deprotonated Cys and Sec were considered because, at a pH of 7.2 (typical of physiological conditions), the fractions of deprotonated Cys and Sec equal 5% and 98%, respectively (calculated with the pKa values of side chains of Cys and Sec, 8.3 and 5.2, respectively). Among these coordinative sites widely diffused in many protein and DNA targets, Sec proteins are found in the viral families Herpesviridae (Epstein–Barr virus, dermatotropic poxvirus [36]), Poxviridae (fowlpox virus [37]), Picornaviridae (Coxsackieviruses B3 and B4 [38]), Flavoridae (Hepatitite C virus [38,39], West nile virus, Japanese encephalitis virus [40]), Filoviridae (Ebola virus [41]), Paramyxoviridae (Measles virus [38]), Retroviridae (HIV-1 [41,42,43], HIV-2 [38], Murine Leukemia virus [36]), and Hepadnaviridae (Hepatitis B [36]). The effects of the environment were considered by performing the calculations in chloroform; indeed, the 4.81 dielectric constant of this solvent is close to the range of 6–7 estimated for a protein environment [44], tentatively assuming the same dielectric constant for either DNA or RNA environments. A preliminary assessment of the possible aquation affecting both KP1019 and SbCl_3_ showed that the substitution of a chloride ligand with one water molecule is thermodynamically disfavored, with calculated Gibbs free energies of aquation > 9 kcal/mol. Thus, we presume that both complexes react with the nucleophilic targets in their administered forms via the substitution of a chloride ligand. The reaction free energy values for the binding of Aubipyc, KP1019, and SbCl_3_ at selected protein or nucleobase sites are reported in Table 2. As shown, Aubipyc and SbCl_3_ displayed similar binding profiles, with highly endergonic coordination at neutral nucleophile sites (Gibbs free energies > 20 kcal/mol), while detecting remarkable exergonic coordination at Cys^−^ and Sec^−^ (Gibbs free energies <20 kcal/mol) (Table 2). Thus, both Aubipyc and SbCl_3_ can be considered as selective protein binders capable of targeting the deprotonated forms of Cys and Sec, which are expected to be formed at high pH, or at specific protein locations inducing a decrease in the pKa values. It should be also noticed that Sec protein sites have lower pKa compared to Cys. Indeed, Sec protein sites are more often found in their deprotonated forms, making them more suitable for metal coordination [45]. On the other hand, the KP1019 complex disclosed a slightly different binding profile compared to Aubipyc and SbCl_3_. Indeed, the exergonic coordination of this complex was detected for Cys^−^, Sec^−^, His, and guanine, while only a slight endergonicity (Gibbs free energies in the range of 0–11 kcal/mol) was detected for coordination at the other nucleophile sites (Table 2). The free energy for the coordination of KP1019 at the neutral Sec was estimated to be less than 1 kcal/mol, thus corresponding to a slightly left-shifted equilibrium. Above all, the ruthenium-based complex KP1019 was less selective towards binding to nucleophilic sites, while Aubipyc and SbCl_3_ showed a well-defined preference for the anionic forms of either Cys or Sec protein sites.

In this frame, our calculations evidenced that the metalation of nucleophilic sites in SARS-CoV-2 proteins or nucleobase strands caused by Aubipyc, SbCl_3_, and KP1019 is possible, although only the deprotonated forms of Cys and Sec residues were found to favorably react with all the three metal complexes. Therefore, the pH of the milieu at which the druggable target is situated, as well as its bulk exposure, are expected to play an utmost important role in determining the occurrence of metalation. In turn, this suggests that the therapeutic efficacy of these metal complexes may be crucially affected by the physico-chemical conditions that are experienced by the SARS-CoV-2 virus.

## 3. Materials and Methods

### 3.1. Preparation of the Metallodrugs Panel

All the tested compounds were well-known inorganic drugs. Auranofin and SbCl_3_ were supplied by Merck (codes A6733 and 215783, respectively; purity ≥ 98%). The iodo-analogue of Auranofin, AuL12, AuOXO6, Aubipyc, NAMI-A, KP1019, and TiCp_2_Cl_2_ were synthesized as reported in the literature with a purity ≥ 95% [6,26,27,28,29,30].

### 3.2. Computational Methods

All calculations were performed with the Gaussian 09 A.02 [46] quantum chemistry package. Geometrical optimizations were carried out in solution by using ωB97X [47], in combination with the basis sets def2SVP for optimization in chloroform, and def2TZVP for the single-point electronic energy evaluations of the optimized structures [48,49]. Frequency calculations were performed to verify the correct nature of the stationary points as well as to estimate zero-point energy (ZPE) and thermal corrections to thermodynamic properties. Indeed, Density-functional Theory DFT gives a good description of geometries and reaction profiles for transition-metal-containing compounds [50,51], including Au- and Ru-based metallodrugs [16,52]. The density functional ωB97X is known to yield accurate geometrical structures and was reported to have reached a high accuracy in the calculation of electronic energies [53,54]. The polarizable continuum model (PCM) using the integral equation formalism variant (IEFPCM) was used to describe the chloroform (ε = 4.81) solvation [55]. For increased accuracy, the experimental values of −74.5 and −104.7 kcal/mol were used for the solvation energies of Cl^−^ and OH^−^ [56].

### 3.3. Cells and Viruses

The SARS-CoV-2 strain, belonging to lineage B.1 (EPI_ISL_2472896), was kindly provided by the Department of Biomedical and Clinical Sciences Luigi Sacco, University of Milan [57]. African green monkey kidney epithelial VERO E6 cell line (ATCC^®^ CRL-1586) was used to propagate and titrate virus stock and to perform the SYRA, adapting a previously published method [58]. Human epithelial lung cancer Calu-3 (ATCC^®^ HTB-55) cell line was used to determine the antiviral activity of candidate compounds in the direct yield reduction assay (DYRA). Both VERO E6 and Calu-3 cell lines have been shown to support SARS-CoV-2 replication [59]. VERO E6 cell line was maintained in high-glucose Dulbecco’s Modified Eagle’s Medium with sodium pyruvate and L-glutamine (DMEM; Euroclone, Milano, Italy), while Calu-3 was maintained in Minimum Essential Medium Eagle (EMEM; Sigma, Darmstadt, Germany) supplemented with 2 mM L-glutamine (L-glut, Euroclone, Milano, Italy). Both culture media were supplemented with 10% Fetal Bovine Serum (FBS; Euroclone, Milano, Italy) and 1% Penicillin/Streptomycin (Pen/Strep, Euroclone, Milano, Italy). The same medium with a lower FBS concentration (1%) was used for the viral propagation and drug susceptibility testing. Cells were incubated at 37 °C in a humidified incubator supplemented with 5% CO_2_. All the virus stocks were titrated by plaque reduction assay (PRA), as previously described [60]. Briefly, VERO E6 cultures were infected with SARS-CoV-2 and monitored by microscopy every 24h. In the presence of large cytopathic effects induced by viral replication, cell cultures were subjected to one cycle of freezing and thawing, with cellular debris then being cleared through centrifugation for 30 min at 1300× *g*, and virus stock titrated through PRA. Viral titer was expressed as plaque-forming units (PFU)/mL.

### 3.4. Drugs and Cytotoxicity Assay

The cytotoxicity of the investigated metal compounds was determined by CellTiter-Glo 2.0 Luminescent Cell Viability Assay (Promega) according to the manufacturer’s protocol. The luminescence values obtained from Calu-3 cells exposed to investigational compounds or dimethyl sulfoxide (DMSO) control for 48h were measured through the GloMax^®^ Discover Multimode Microplate Reader (Promega, Madison, WI, USA) and elaborated with the GraphPad PRISM software version 6.01 (La Jolla, San Diego, CA, USA) to calculate the CC_50_ and the CC_20_. Remdesivir (MCE^®^, Monmouth Junction, NJ, USA, cat. HY-104077), used as reference compound, was supplied as powder, and dissolved in 100% DMSO.

### 3.5. Antiviral Assays

To determine the antiviral activity of candidate compounds against SARS-CoV-2, a DYRA, based on the infection of cells in the presence of serial drug dilutions, was performed as previously described, with minor modifications [25]. Briefly, 25,000 Calu-3, pre-seeded in the 96-well plates, were treated with serial dilutions of each tested compound, and incubated for 30′ at 37 °C with 5% CO_2_. The virus stock was added at a concentration of 250 PFU/well, then, after 1 h of adsorption, the medium was removed, and fresh dilutions of each tested compound were added to the cells. After an incubation of 48h at 37 °C with 5% CO_2_, the antiviral activity was measured on the cell monolayers by an immunodetection assay (IA), consisting of the fixation and permeabilization of cells, followed by 1 h incubation with a monoclonal SARS Nucleocapsid Protein Antibody (Novus, Milano, Italy, cat. AP201054), diluted 1:1000 in blocking buffer (PBS containing 1% BSA and 0.1% Tween 20) [61]. After washing, monolayers were incubated for 1 h with a polyclonal HRP-coupled anti-mouse IgG secondary antibody (Novus Bio, Milano, Italy, NB7570), diluted 1:5000 in blocking buffer. After cell washing, the 3,3′,5,5′-Tetramethylbenzidine substrate (Sigma Aldrich, Darmstadt, Germany) was added to each well and the reaction was stopped with one volume of 0.5 M sulfuric acid. Absorbance was measured at 450 nm optical density (OD450) using the Absorbance Module of the GloMax^®^ Discover Multimode Microplate Reader (Promega).

Compounds not active in DYRA were then analyzed in SYRA to characterize late antiviral effects. SYRA was performed, adapting to SARS-CoV-2 a protocol already published [61]. Supernatants containing viral particles produced during DYRA were briefly harvested from each well, diluted, and used to infect pre-seeded 10,000 VERO E6 cells. After 1 h of adsorption, viral supernatants were removed, fresh medium was added, and cells were incubated for 24 h at 37 °C with 5% CO_2_. The IA was performed on the cell monolayers as described above. The half-maximal inhibitory concentration (IC_50_) was calculated through a non-linear regression analysis of the dose–response curves generated with GraphPad PRISM software version 6.01. In each test, Remdesivir was used as a reference compound against SARS-CoV-2. Infected and uninfected cells without drugs were used to calculate the 100% and 0% of viral replication, respectively. Selectivity Index (SI) was calculated as the ratio between CC_50_ and IC_50_. In principle, the higher the SI value, the more efficacy and safety should be observed during in vivo treatment.

## 4. Conclusions

Metal compounds offer a rich variety of chemical structures and reactivities that merit to be considered in the screening libraries of chemical substances for new drug discovery [23]. Indeed, drug repurposing is a time-saving and cost-efficient approach for speeding up the process of the clinical evaluation of candidate drugs against novel diseases, such as COVID-19 [62].

There are already some good indications in the literature that a few metal compounds might perform reasonably well in the treatment of COVID-19. This observation led us to expand this kind of study and to prepare a small panel of metal compounds to be tested as potential anti-SARS-CoV-2 agents. The screening revealed that three out of the nine metallodrugs belonging to the panel, i.e., Aubipic, KP1019 and antimony chloride, possess promising properties against SARS-CoV-2, although the low selectivity index suggests that these molecules are not the most appropriate for in vivo testing.

Based on the previous literature available on these metal-based drugs, it is highly probable that these compounds characterized by very soft metal centers may interact preferentially with selected residues of proteins [6,63,64,65]. A computational study revealed that these compounds indeed manifest a good selectivity for thiol and selenol groups of proteins. The affinity is much greater when thiols and selenols exist in their deprotonated anionic form. These considerations may help the search for the actual biomolecular targets for the above mentioned metallodrugs. Such mechanistic information may turn out to be useful in the design and development of improved metallodrugs against SARS-CoV-2.

## Figures and Tables

**Table 1:** Cytotoxicity and antiviral activity of metallic compounds. All compounds were tested by the direct yield reduction assay (DYRA) protocols. The compounds not active in DYRA were subsequently screened using the secondary yield reduction assay (SYRA) to evaluate possible activity in the late phases of viral replication not detectable with DYRA.

Compound	CC_50_ (µM) ^1^	IC_50_ (µM) ^2^	Selectivity Index ^5^
Auranofin	3.7	Not active ^3^	---
Au(Pet_3_)I	12	Not active ^3^	---
AuL12	19	Not active ^3^	---
AuOXO6	40	Not active ^3^	---
Aubipyc	67	6.3 ± 3.1	10.6
NAMI-A	>200	Not active ^3^	---
KP1019	60	8.8 ± 1.5	6.8
SbCl_3_	200	31.1 ± 15.3	6.4
TiCp_2_Cl_2_	>200	47.3 ± 1.4 ^4^	>4.2
Remdesivir	97	0.2 ± 0.05	485

^1^ CC_50_: half-maximal cytotoxic concentration; ^2^ IC50: half-maximal inhibitory concentration; ^3^ compound not active with both DYRA and SYRA. ^4^ Determined with SYRA while not active in DYRA. ^5^ Calculated as the CC_50_/IC_50_ ratio. When not soluble in water, DMSO was used to solubilize compounds. The use of organic solvent was kept as low as possible (<1%) and the relative blank sample was used to avoid bias.

**Table 2:** Gibbs free energies for the binding of the selected metallodrugs at possible target nucleophile sites in SARS-CoV-2 proteins or nucleobase strands via the substitution of a chloride ligand. All values are reported in kcal/mol.

Nucleophiles	Aubipyc	KP1019	SbCl_3_
Cys	52.4	10.6	42.6
Cys^−^	−23.8	−32.0	−36.1
Sec	49.1	0.4	40.4
Sec^−^	−27.8	−24.4	−30.4
His (chain at position 1)	37.0	−10.1	21.2
His (chain at position 2)	37.5	−12.6	19.0
Guanine	34.1	−7.8	25.0
Adenine	50.4	11.4	31.0
Water	68.0	9.1	44.7

## References

[1] Hu B., Guo H., Zhou P., Shi Z.-L. (2020). Characteristics of SARS-CoV-2 and COVID-19. Nat. Rev. Microbiol..

[2] Shah A.S., Gribben C., Bishop J., Hanlon P., Caldwell D., Wood R., Reid M., McMenamin J., Goldberg D., Stockton D. (2021). Effect of Vaccination on Transmission of SARS-CoV-2. N. Engl. J. Med..

[3] Tarighi P., Eftekhari S., Chizari M., Sabernavaei M., Jafari D., Mirzabeigi P. (2021). A review of potential suggested drugs for coronavirus disease (COVID-19) treatment. Eur. J. Pharmacol..

[4] Fischer W., Eron J.J., Holman W., Cohen M.S., Fang L., Szewczyk L.J., Sheahan T.P., Baric R., Mollan K.R., Wolfe C.R. (2021). Molnupiravir, an Oral Antiviral Treatment for COVID-19. medRxiv.

[5] Cirri D., Bartoli F., Pratesi A., Baglini E., Barresi E., Marzo T. (2021). Strategies for the Improvement of Metal-Based Chemotherapeutic Treatments. Biomedicines.

[6] Marzo T., Mendola D. (2021). La Strike a Balance: Between Metals and Non-Metals, Metalloids as a Source of Anti-Infective Agents. Inorganics.

[7] Anthony E.J., Bolitho E.M., Bridgewater H.E., Carter O.W.L., Donnelly J.M., Imberti C., Lant E.C., Lermyte F., Needham R.J., Palau M. (2020). Metallodrugs are unique: Opportunities and challenges of discovery and development. Chem. Sci..

[8] Barry N.P.E., Sadler P.J. (2013). Exploration of the medical periodic table: Towards new targets. Chem. Commun..

[9] Marzo T., Cirri D., Pollini S., Prato M., Fallani S., Cassetta M.I., Novelli A., Rossolini G.M., Messori L. (2018). Auranofin and its Analogues Show Potent Antimicrobial Activity against Multidrug-Resistant Pathogens: Structure-Activity Relationships. ChemMedChem.

[10] Miodragović D., Merlino A., Swindell E.P., Bogachkov A., Ahn R.W., Abuhadba S., Ferraro G., Marzo T., Mazar A.P., Messori L. (2019). Arsenoplatin-1 Is a Dual Pharmacophore Anticancer Agent. J. Am. Chem. Soc..

[11] Mjos K.D., Orvig C. (2014). Metallodrugs in medicinal inorganic chemistry. Chem. Rev..

[12] Miranda V.M. (2021). Medicinal inorganic chemistry: An updated review on the status of metallodrugs and prominent metallodrug candidates. Rev. Inorg. Chem..

[13] Sun H., Zhang Q., Wang R., Wang H., Wong Y.T., Wang M., Hao Q., Yan A., Kao R.Y.T., Ho P.L. (2020). Resensitizing carbapenem- and colistin-resistant bacteria to antibiotics using auranofin. Nat. Commun..

[14] Pearson R.G. (1995). The HSAB Principle—More quantitative aspects. Inorganica Chim. Acta.

[15] Zoppi C., Messori L., Pratesi A. (2020). ESI MS studies highlight the selective interaction of Auranofin with protein free thiols. Dalt. Trans..

[16] Tolbatov I., Cirri D., Marchetti L., Marrone A., Coletti C., Re N., La Mendola D., Messori L., Marzo T., Gabbiani C. (2020). Mechanistic Insights Into the Anticancer Properties of the Auranofin Analog Au(PEt3)I: A Theoretical and Experimental Study. Front. Chem..

[17] Fabbrini M.G., Cirri D., Pratesi A., Ciofi L., Marzo T., Guerri A., Nistri S., Dell’Accio A., Gamberi T., Severi M. (2018). A Fluorescent Silver(I) Carbene Complex with Anticancer Properties: Synthesis, Characterization, and Biological Studies. ChemMedChem.

[18] Yuan S., Wang R., Chan J.F.-W., Zhang A.J., Cheng T., Chik K.K.-H., Ye Z.-W., Wang S., Lee A.C.-Y., Jin L. (2020). Metallodrug ranitidine bismuth citrate suppresses SARS-CoV-2 replication and relieves virus-associated pneumonia in Syrian hamsters. Nat. Microbiol..

[19] Gil-Moles M., Basu U., Büssing R., Hoffmeister H., Türck S., Varchmin A., Ott I. (2020). Gold Metallodrugs to Target Coronavirus Proteins: Inhibitory Effects on the Spike-ACE2 Interaction and on PLpro Protease Activity by Auranofin and Gold Organometallics. Chemistry.

[20] Griffith D.M., Li H., Werrett M.V., Andrews P.C., Sun H. (2021). Medicinal chemistry and biomedical applications of bismuth-based compounds and nanoparticles. Chem. Soc. Rev..

[21] Yang N., Tanner J.A., Zheng B.J., Watt R.M., He M.L., Lu L.Y., Jiang J.Q., Shum K.T., Lin Y.P., Wong K.L. (2007). Bismuth complexes inhibit the SARS coronavirus. Angew. Chem. Int. Ed..

[22] Marzo T., Messori L. (2020). A Role for Metal-Based Drugs in Fighting COVID-19 Infection? The Case of Auranofin. ACS Med. Chem. Lett..

[23] Cirri D., Pratesi A., Marzo T., Messori L. (2021). Metallo therapeutics for COVID-19. Exploiting metal-based compounds for the discovery of new antiviral drugs. Expert Opin. Drug Discov..

[24] Gil-Moles M., Türck S., Basu U., Pettenuzzo A., Bhattacharya S., Rajan A., Ma X., Büssing R., Wölker J., Burmeister H. (2021). Metallodrug Profiling against SARS-CoV-2 Target Proteins Identifies Highly Potent Inhibitors of the S/ACE2 interaction and the Papain-like Protease PL^pro^. Chemistry.

[25] Martina M.G., Vicenti I., Bauer L., Crespan E., Rango E., Boccuto A., Olivieri N., Incerti M., Zwaagstra M., Allodi M. (2021). Bithiazole Inhibitors of Phosphatidylinositol 4-Kinase (PI4KIIIβ) as Broad-Spectrum Antivirals Blocking the Replication of SARS-CoV-2, Zika Virus, and Human Rhinoviruses. ChemMedChem.

[26] Marzo T., Cirri D., Gabbiani C., Gamberi T., Magherini F., Pratesi A., Guerri A., Biver T., Binacchi F., Stefanini M. (2017). Auranofin, Et3PAuCl, and Et3PAuI Are Highly Cytotoxic on Colorectal Cancer Cells: A Chemical and Biological Study. ACS Med. Chem. Lett..

[27] Gorini G., Magherini F., Fiaschi T., Massai L., Becatti M., Modesti A., Messori L., Gamberi T. (2021). Au2phen and Auoxo6, Two Dinuclear Oxo-Bridged Gold(III) Compounds, Induce Apoptotic Signaling in Human Ovarian A2780 Cancer Cells. Biomedicines.

[28] Tomasello M.F., Nardon C., Lanza V., Di Natale G., Pettenuzzo N., Salmaso S., Milardi D., Caliceti P., Pappalardo G., Fregona D. (2017). New comprehensive studies of a gold(III) Dithiocarbamate complex with proven anticancer properties: Aqueous dissolution with cyclodextrins, pharmacokinetics and upstream inhibition of the ubiquitin-proteasome pathway. Eur. J. Med. Chem..

[29] Alessio E., Messori L. (2019). NAMI-A and KP1019/1339, Two Iconic Ruthenium Anticancer Drug Candidates Face-to-Face: A Case Story in Medicinal Inorganic Chemistry. Molecules.

[30] Ellahioui Y., Prashar S., Gómez-Ruiz S. (2017). Anticancer Applications and Recent Investigations of Metallodrugs Based on Gallium, Tin and Titanium. Inorganics.

[31] Rothan H.A., Stone S., Natekar J., Kumari P., Arora K., Kumar M. (2020). The FDA-approved gold drug auranofin inhibits novel coronavirus (SARS-COV-2) replication and attenuates inflammation in human cells. Virology.

[32] Zhang L., Lin D., Sun X., Curth U., Drosten C., Sauerhering L., Becker S., Rox K., Hilgenfeld R. (2020). Crystal structure of SARS-CoV-2 main protease provides a basis for design of improved α-ketoamide inhibitors. Science.

[33] Ghosh A.K., Brindisi M., Shahabi D., Chapman M., Mesecar A.D. (2020). Drug Development and Medicinal Chemistry Efforts toward SARS-Coronavirus and Covid-19 Therapeutics. ChemMedChem.

[34] Jia Z., Yan L., Ren Z., Wu L., Wang J., Guo J., Zheng L., Ming Z., Zhang L., Lou Z. (2019). Delicate structural coordination of the Severe Acute Respiratory Syndrome coronavirus Nsp13 upon ATP hydrolysis. Nucleic Acids Res..

[35] Gui M., Song W., Zhou H., Xu J., Chen S., Xiang Y., Wang X. (2017). Cryo-electron microscopy structures of the SARS-CoV spike glycoprotein reveal a prerequisite conformational state for receptor binding. Cell Res..

[36] Taylor E.W., Nadimpalli R.G., Ramanathan C.S. (1997). Genomic structures of viral agents in relation to the biosynthesis of selenoproteins. Biol. Trace Elem. Res..

[37] Mix H., Lobanov A.V., Gladyshev V.N. (2007). SECIS elements in the coding regions of selenoprotein transcripts are functional in higher eukaryotes. Nucleic Acids Res..

[38] Zhang W., Ramanathan C.S., Nadimpalli R.G., Bhat A.A., Cox A.G., Taylor E.W. (1999). Selenium-dependent glutathione peroxidase modules encoded by RNA viruses. Biol. Trace Elem. Res..

[39] Zhang W., Cox A.G., Taylor E.W. (1999). Hepatitis C virus encodes a selenium-dependent glutathione peroxidase gene. Medizinische Klin..

[40] Zhong H., Taylor E.W. (2004). Structure and dynamics of a predicted ferredoxin-like selenoprotein in Japanese encephalitis virus. J. Mol. Graph. Model..

[41] Taylor E.W., Ruzicka J.A., Premadasa L., Zhao L. (2016). Cellular Selenoprotein mRNA Tethering via Antisense Interactions with Ebola and HIV-1 mRNAs May Impact Host Selenium Biochemistry. Curr. Top. Med. Chem..

[42] Zhao L., Cox A.G., Ruzicka J.A., Bhat A.A., Zhang W., Taylor E.W. (2000). Molecular modeling and in vitro activity of an HIV-1-encoded glutathione peroxidase. Proc. Natl. Acad. Sci. USA.

[43] Cohen I., Boya P., Zhao L., Métivier D., Andreau K., Perfettini J.L., Weaver J.G., Badley A., Taylor E.W., Kroemer G. (2004). Anti-apoptotic activity of the glutathione peroxidase homologue encoded by HIV-1. Apoptosis.

[44] Li L., Li C., Zhang Z., Alexov E. (2013). On the Dielectric “Constant” of Proteins: Smooth Dielectric Function for Macromolecular Modeling and Its Implementation in DelPhi. J. Chem. Theory Comput..

[45] Tolbatov I., Marrone A., Paciotti R., Re N., Coletti C. (2021). Multilayered Modelling of the Metallation of Biological Targets. Proceedings of the International Conference on Computational Science and Its Applications.

[46] Citation|Gaussian.com. https://gaussian.com/citation/.

[47] Chai J.-D., Head-Gordon M. (2008). Systematic optimization of long-range corrected hybrid density functionals. J. Chem. Phys..

[48] Andrae D., Häußermann U., Dolg M., Stoll H., Preuß H. (1991). Energy-adjustedab initio pseudopotentials for the second and third row transition elements: Molecular test for M2 (M=Ag, Au) and MH (M=Ru, Os). Theor. Chim. Acta.

[49] Weigend F., Ahlrichs R. (2005). Balanced basis sets of split valence, triple zeta valence and quadruple zeta valence quality for H to Rn: Design and assessment of accuracy. Phys. Chem. Chem. Phys..

[50] Tolbatov I., Marzo T., Coletti C., La Mendola D., Storchi L., Re N., Marrone A. (2021). Reactivity of antitumor coinage metal-based N-heterocyclic carbene complexes with cysteine and selenocysteine protein sites. J. Inorg. Biochem..

[51] Tolbatov I., Marzo T., Cirri D., Gabbiani C., Coletti C., Marrone A., Paciotti R., Messori L., Re N. (2020). Reactions of cisplatin and cis-[PtI 2(NH 3) 2] with molecular models of relevant protein sidechains: A comparative analysis. J. Inorg. Biochem..

[52] Barresi E., Tolbatov I., Pratesi A., Notarstefano V., Baglini E., Daniele S., Taliani S., Re N., Giorgini E., Martini C. (2020). A mixed-valence diruthenium(ii,iii) complex endowed with high stability: From experimental evidence to theoretical interpretation. Dalt. Trans..

[53] Dohm S., Hansen A., Steinmetz M., Grimme S., Checinski M.P. (2018). Comprehensive Thermochemical Benchmark Set of Realistic Closed-Shell Metal Organic Reactions. J. Chem. Theory Comput..

[54] Tolbatov I., Coletti C., Marrone A., Re N. (2020). Reactivity of arsenoplatin complex versus water and thiocyanate: A DFT benchmark study. Theor. Chem. Acc..

[55] Tomasi J., Mennucci B., Cancès E. (1999). The IEF version of the PCM solvation method: An overview of a new method addressed to study molecular solutes at the QM ab initio level. J. Mol. Struct. THEOCHEM.

[56] Kelly C.P., Cramer C.J., Truhlar D.G. (2006). Single-Ion Solvation Free Energies and the Normal Hydrogen Electrode Potential in Methanol, Acetonitrile, and Dimethyl Sulfoxide. J. Phys. Chem. B.

[57] Lai A., Bergna A., Caucci S., Clementi N., Vicenti I., Dragoni F., Cattelan A.M., Menzo S., Pan A., Callegaro A. (2020). Molecular Tracing of SARS-CoV-2 in Italy in the First Three Months of the Epidemic. Viruses.

[58] Vicenti I., Martina M.G., Boccuto A., De Angelis M., Giavarini G., Dragoni F., Marchi S., Trombetta C.M., Crespan E., Maga G. (2021). System-oriented optimization of multi-target 2,6-diaminopurine derivatives: Easily accessible broad-spectrum antivirals active against flaviviruses, influenza virus and SARS-CoV-2. Eur. J. Med. Chem..

[59] Chu H., Chan J.F.-W., Yuen T.T.-T., Shuai H., Yuan S., Wang Y., Hu B., Yip C.C.-Y., Tsang J.O.-L., Huang X. (2020). Comparative tropism, replication kinetics, and cell damage profiling of SARS-CoV-2 and SARS-CoV with implications for clinical manifestations, transmissibility, and laboratory studies of COVID-19: An observational study. Lancet Microbe.

[60] Vicenti I., Boccuto A., Giannini A., Dragoni F., Saladini F., Zazzi M. (2018). Comparative analysis of different cell systems for Zika virus (ZIKV) propagation and evaluation of anti-ZIKV compounds in vitro. Virus Res..

[61] Vicenti I., Dragoni F., Giannini A., Giammarino F., Spinicci M., Saladini F., Boccuto A., Zazzi M. (2020). Development of a Cell-Based Immunodetection Assay for Simultaneous Screening of Antiviral Compounds Inhibiting Zika and Dengue Virus Replication. SLAS Discov..

[62] Parvathaneni V., Gupta V. (2020). Utilizing drug repurposing against COVID-19—Efficacy, limitations, and challenges. Life Sci..

[63] Messori L., Scaletti F., Massai L., Cinellu M.A., Gabbiani C., Vergara A., Merlino A. (2013). The mode of action of anticancer gold-based drugs: A structural perspective. Chem. Commun..

[64] Casini A., Hartinger C., Gabbiani C., Mini E., Dyson P.J., Keppler B.K., Messori L. (2008). Gold(III) compounds as anticancer agents: Relevance of gold–protein interactions for their mechanism of action. J. Inorg. Biochem..

[65] Rilak Simović A., Masnikosa R., Bratsos I., Alessio E. (2019). Chemistry and reactivity of ruthenium(II) complexes: DNA/protein binding mode and anticancer activity are related to the complex structure. Coord. Chem. Rev..

